# Antibiotic monensin synergizes with EGFR inhibitors and oxaliplatin to suppress the proliferation of human ovarian cancer cells

**DOI:** 10.1038/srep17523

**Published:** 2015-12-07

**Authors:** Youlin Deng, Junhui Zhang, Zhongliang Wang, Zhengjian Yan, Min Qiao, Jixing Ye, Qiang Wei, Jing Wang, Xin Wang, Lianggong Zhao, Shun Lu, Shengli Tang, Maryam K. Mohammed, Hao Liu, Jiaming Fan, Fugui Zhang, Yulong Zou, Junyi Liao, Hongbo Qi, Rex C. Haydon, Hue H. Luu, Tong-Chuan He, Liangdan Tang

**Affiliations:** 1 Department of Obstetrics and Gynecology, and Physical Examination, the First Affiliated Hospital of Chongqing Medical University, Chongqing 400046, China; 2 Molecular Oncology Laboratory, Department of Orthopaedic Surgery and Rehabilitation Medicine, The University of Chicago Medical Center, Chicago, IL 60637, USA; 3 Ministry of Education Key Laboratory of Diagnostic Medicine, and the Affiliated Hospitals of Chongqing Medical University, Chongqing 400016, China; 4 School of Bioengineering, Chongqing University, Chongqing 400044, China; 5 Department of Surgery, West China Hospital of Sichuan University, Chengdu 610041, China; 6 Department of Orthopaedic Surgery, the Second Affiliated Hospital of Lanzhou University, Lanzhou, Gansu 730000, China; 7 Department of Orthopaedic Surgery, Shandong Provincial Hospital and Shandong University School of Medicine, Jinan 250012, China; 8 Departments of General Surgery, the Affiliated Zhongnan Hospital of Wuhan University, Wuhan 430071, China

## Abstract

Ovarian cancer is the most lethal gynecologic malignancy with an overall cure rate of merely 30%. Most patients experience recurrence within 12–24 months of cure and die of progressively chemotherapy-resistant disease. Thus, more effective anti-ovarian cancer therapies are needed. Here, we investigate the possibility of repurposing antibiotic monensin as an anti-ovarian cancer agent. We demonstrate that monensin effectively inhibits cell proliferation, migration and cell cycle progression, and induces apoptosis of human ovarian cancer cells. Monensin suppresses multiple cancer-related pathways including Elk1/SRF, AP1, NFκB and STAT, and reduces EGFR expression in ovarian cancer cells. Monensin acts synergistically with EGFR inhibitors and oxaliplatin to inhibit cell proliferation and induce apoptosis of ovarian cancer cells. Xenograft studies confirm that monensin effectively inhibits tumor growth by suppressing cell proliferation through targeting EGFR signaling. Our results suggest monensin may be repurposed as an anti-ovarian cancer agent although further preclinical and clinical studies are needed.

Ovarian cancer is the fifth most common cancer in women in the United States and the most lethal gynecologic malignancy^1^,^2^. Efforts at early detection and new therapeutic approaches to reduce mortality have been met with limited clinical successes, in part because the origin and pathogenesis of epithelial ovarian cancer are poorly understood^2^. Although epithelial ovarian cancer (EOC) is the most common subtype, increasing evidence indicates that EOC itself is composed of a diverse group of tumors that can be further classified on the basis of distinctive morphologic and genetic features^1^,^2^,^3^,^4^,^5^. Given the absence of an effective screening strategy, a mere 20% of ovarian cancers are diagnosed while confined to the ovaries. Over the past two decades, the 5-year survival rate for ovarian cancer patients has substantially improved, largely due to improved surgical techniques and empirically optimized chemotherapy regimens of cytotoxic platinum-combination drugs. In spite of this improvement, the overall cure rate remains approximately 30%^1^,^6^. Most patients experience recurrence within 12–24 months and die of progressively chemotherapy-resistant disease^1^,^6^.

Given the heterogeneity of human ovarian cancers, significant improvements in long-term survival may hinge on translating recent insights into the molecular and cellular characteristics of ovarian cancers into personalized treatment strategies, optimizing methods of screening or early detection, and developing novel therapeutics. While significant progress has recently been made in the development of novel targeted therapies for human cancers, including ovarian cancers^1^,^3^,^4^,^5^, an effective alternative to drug development is repurposing drugs. Several examples of such drugs are currently in various stages of clinical trials^7^,^8^.

In this study, we investigate the anti-cancer activity of the antibiotic monensin against human ovarian cancer cells. Monensin (aka., Rumensin) is a polyether ionophore antibiotic secreted by the bacteria *Streptomyces cinnamonensis*^9^,^10^. Earlier studies have demonstrated that monensin exhibits cytotoxic effects against several types of cancer cells^10^,^11^,^12^,^13^,^14^,^15^,^16^,^17^,^18^. However, it remains unclear if monensin exerts similar anticancer effects on human ovarian cancer cells. Using commonly-utilized human ovarian cancer lines HeyA8 and SKOV3, we have demonstrated that monensin effectively inhibits cell proliferation, cell migration, and cell cycle progression, and induces apoptosis of human ovarian cancer cells. Monensin was shown to target multiple cancer-related signaling pathways including Elk1/SRF, AP1, NFκB and STAT, and suppress the expression of EGFR, but not IGF-1R, in ovarian cancer cells. Further analysis indicated that monensin acts synergistically with EGFR inhibitors and the chemotherapeutic drug oxaliplatin to inhibit cell proliferation and induce apoptosis of human ovarian cancer cells. In *in vivo* xenograft studies, monensin effectively inhibited xenograft tumor growth, perhaps by inhibiting cell proliferation through targeting EGFR signaling. Therefore, our results strongly suggest that monensin has potential to be repurposed as an anti-ovarian cancer agent. Future studies should be directed towards testing monensin’s anti-cancer efficacy in preclinical and clinical studies.

## Results

### Monensin effectively inhibits cell proliferation and migration of human ovarian cancer cells

**Figure 1:**
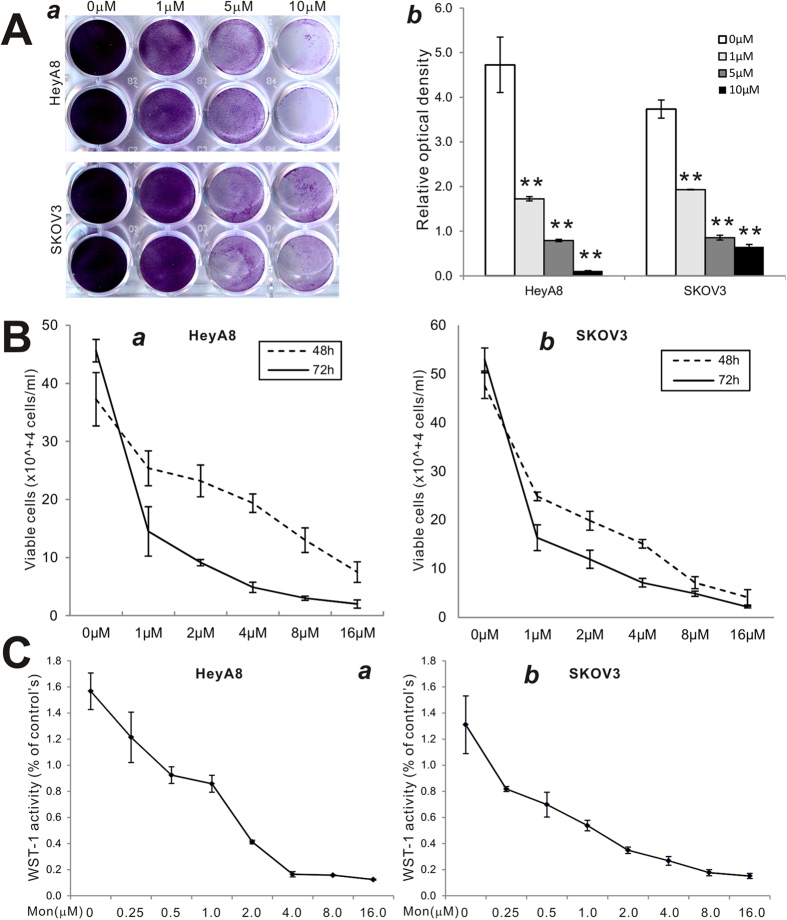
Monensin effectively inhibits the proliferation of human ovarian cancer cells. (**A**) Crystal violet staining assay. Subconfluent HeyA8 and SKOV3 cells were seeded in 12-well plates and treated with monensin at the indicated concentrations. At 72 h post treatment, the cells were fixed and stained with crystal violet (*a*). Crystal violet stain was dissolved and measured quantitatively for optical absorbance (*b*). **p < 0.001. **(B**) Viable cell counting assay. Subconfluent HeyA8 (*a*) and SKOV3 (*b*) cells were seeded in 12-well plates and treated with monensin at the indicated concentrations. At 48 h and 72 h post treatment, the viable cells were collected, stained with trypan blue and counted under a bright field microscope. (**C**) WST-1 cell proliferation assay. Subconfluent HeyA8 (*a*) and SKOV3 (*b*) cells were seeded in 96-well plates and treated with monensin at the indicated concentrations. At 24 h post treatment, the WST-1 reagent (BD Bioscience) was added to plates and incubated for 1 h and absorbance measurement was performed. All assay conditions were done in triplicate.

We sought to test the effect of the antibiotic monensin on the proliferative activity of two commonly-used human ovarian cancer lines HeyA8 and SKOV3. Sub-confluent HeyA8 and SKOV3 cells were grown in increasing concentrations of monensin. Crystal violet staining results indicated that monensin effectively inhibited cell proliferation in both cell lines at concentrations as low as 1 μM, and completely inhibited cell proliferation at 10 μM (Fig. 1A**, panel a**), especially in HeyA8 cells. This was confirmed by quantitative analysis of crystal violet staining data (p < 0.001 at all three monensin concentrations) (Fig. 1A**, panel b**). We also conducted direct cell counting after exponentially growing HeyA8 and SKOV3 cells were treated with varying concentrations of monensin (0 μM to 16 μM). We found that the number of viable cells decreased significantly when the concentration of monensin increased in both cell lines at both examined time points (p < 0.001) (Fig. 1B**, panels a,b**). Further evaluation of anti-proliferative effects was accomplished with the more sensitive and quantitative WST-1 proliferation assay, which found that statistically significant inhibition of cell proliferation occurred at concentrations as low as 0.25 μM monensin in HeyA8 (p < 0.05) and SKOV3 (p < 0.001) (Fig. 1C**, panels a,b**). Taken together, our results from these cell proliferation assays demonstrate that monensin can effectively inhibit the cell proliferation of ovarian cancer cells.

**Figure 2:**
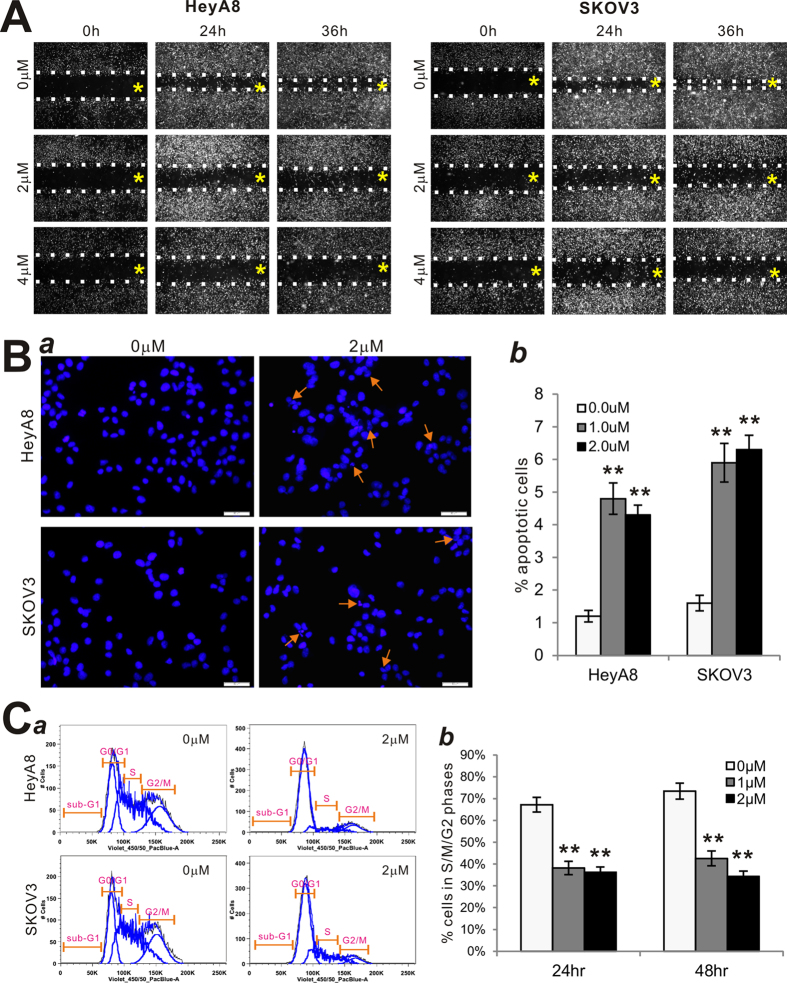
Monensin inhibits cell wounding healing and induces apoptosis of human ovarian cancer cells. (**A**) Cell wounding assay. Freshly subconfluent HeyA8 and SKOV3 cells were wounded with micro-pipette tips and treated with monensin at the indicated concentrations. The wounding gaps were recorded at 0 h, 24 h and 36 h after monensin treatments. The yellow “*” signs indicate the reference points for imaging, while the dotted lines indicate the fronts of cell wounding. Each assay condition was done in triplicate. (**B**) Hoechst 33258 staining assay. Subconfluent HeyA8 and SKOV3 cells were treated with 1 or 2 μM monensin or solvent control. At 24 h post treatment, cells were collected, fixed and stained with Hoechst 33258 and examined under a fluorescence microscope (*a*). Apparent apoptotic cells were counted in at least 10 random fields under 100× magnification (*b*). **p< 0.01 (monensin treated vs. control group). **(C)** Cell cycle analysis. Subconfluent HeyA8 and SKOV3 cells were treated with monensin or vehicle control for 24 h or 48 h. Cells were collected, fixed, stained with Hoechst 33258, and subjected to FACS analysis (*a*). Percentages of cells in non-G1 phase were tabulated and graphed (*b*). Each assay condition was done in triplicate. **p< 0.01 (monensin treated vs. control group).

We next examined if monensin exerts any effect on cell migration and wound healing in ovarian cancer cells. Freshly confluent HeyA8 and SKOV3 monolayer cells were wounded and treated with 0, 2, or 4 μM monensin. The width of the wound defect, relative to the starting width, was measured at 32% and 11% for Hey8 cells at 24 h and 36 h, respectively, and 27% and 8% for SKOV3 cells at 24 h and 36 h, respectively (Fig. 2A). However, in the presence of 2 and 4 μM monensin, the rate of gap closure was significantly reduced. Specifically, with 2 μM monensin, the defect was approximately 75% and 68% at 24 h and 36 h in HeyA8 cells, respectively, compared to that at 0 h. Similar percentages of gap closure were obtained in SKOV3 cells (Fig. 2A). The rate of gap closures was further decreased when cells were exposed to 4 μM monensin. Approximately 80% of the gap remained open in both cell lines (Fig. 2A). These results suggest that monensin inhibits cell migration and cell wounding healing of ovarian cancer cells in a dose-dependent fashion.

### Monensin induces apoptosis and inhibits cell cycle progression in human ovarian cancer cells

To understand the possible mechanisms underlying monensin-induced inhibition of cell proliferation, we investigated if monensin can induce apoptosis in ovarian cancer cells. When exponentially proliferating HeyA8 and SKOV3 cells were treated with 0 μM, 1 μM, or 2 μM monensin for 24 h and stained with Hoechst 33258, significant numbers of apoptotic cells were observed (Fig. 2B**, panel a**). Quantitative analysis indicated that the percentages of apoptotic cells were significantly increased in monensin treated HeyA8 and SKOV3 cells (p < 0.01) (Fig. 2B**, panel b**). We conducted cell cycle analysis on monensin-treated cells and found a significant increase in cells arrested in G1 phase, as well as decreased cells in S/M phase in monensin-treated HeyA8 and SKOV3 cells relative to the controls (p < 0.001) (Fig. 2C**, panels a,b**). These results suggest that monensin’s inhibition of ovarian cancer cell proliferation may be due in part to induction of apoptosis and inhibition of cell cycle progression.

### Monensin inhibits multiple cancer-related signaling pathways, including the downstream effectors of EGFR signaling

**Figure 3:**
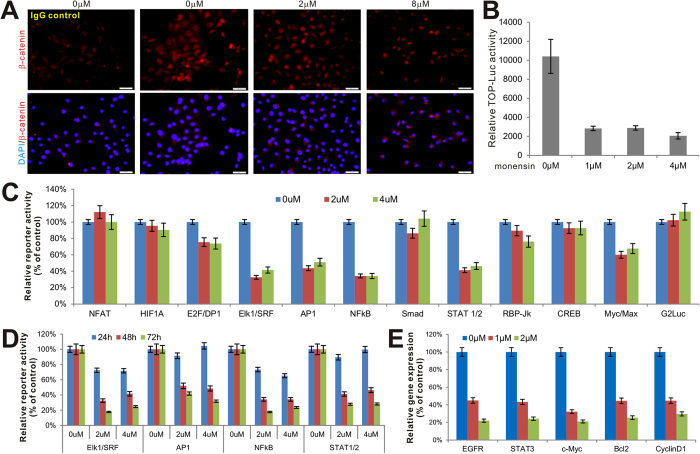
Monensin inhibits multiple cancer-associated signaling pathways, including downstream mediators of EGFR signaling. (**A**) Effect of monensin on intracellular β-catenin level. Subconfluent SKOV3 cells were first transduced with Ad-Wnt3A or AdGFP for 16 h, and treated with varied concentrations of monensin for additional 36 h. Cells were fixed and subjected immunofluorescence staining with an anti-β-catenin antibody. The cell nuclei were counterstained with DAPI. Control IgG was used as a negative control. Representative results are shown. (**B**) Monensin inhibits Tcf/Lef reporter activity. Subconfluent SKOV3 cells were transfected with TOP-Luc reporter plasmid and infected with Ad-Wnt3A or AdGFP for 16 h, followed by a treatment with varied concentrations of monensin for 48 h. Cells were lysed and subjected to luciferase activity assays using Promega’s firefly Luciferase Assay System. Each assay condition was done in triplicate. (**C**) Effect of monensin on the 11 cancer-associated pathway reporter activities. Subconfluent SKOV3 cells were transfected with the homemade Gaussia luciferase reporters for the 11 cancer-associated pathways and a constitutively active reporter pG2Luc. At 16 h post transfection the cells were treated with varied concentrations of monensin for additional 48 h. the culture medium was collected for Gaussia luciferase activity assay using BioLux Gaussia Luciferase Assay Kit (New England Biolabs). Each assay condition was done in triplicate. (**D**) Monensin inhibits four pathways in dose- and time-dependent manners. The selected four pathway reporters were transfected into SKOV3 cells as described in (**C**), except that Gaussia luciferase activities were measured at 24 h, 48 h and 72 h post treatment. **(E)** Monensin inhibits the expression of genes involved in cell proliferation. Subconfluent SKOV3 cells were treated with the indicated concentrations of monensin for 48 h. Total RNA was isolated and subjected to qPCR analysis of the expression of the indicated genes. Human GAPDH was used as the reference gene.

A recent study suggests that monensin may exert anti-cancer activity in colorectal cancer by inhibiting Wnt/β-catenin activity^18^. To evaluate if a similar effect occurs in ovarian cancer cells, we used exogenous Wnt3A to activate the canonical Wnt pathway and assessed the effect of monensin on β-catenin signaling activity via nuclear staining. When SKOV3 cells were stimulated with Ad-Wnt3A, we observed a remarkable elevation of nuclear β-catenin staining, which was significantly inhibited by monensin in a dose-dependent manner (Fig. 3A). Accordingly, the Wnt3A-activated Tcf/β-catenin reporter activity in SKOV3 cells was effectively inhibited by monensin at concentrations as low as 1 μM (Fig. 3B). Similar results were obtained using HeyA8 cells (data not shown). These results confirm that monensin can inhibit the Wnt/β-catenin signaling pathway in ovarian cancer cells as previously demonstrated in a colorectal cancer cell line.

Although it has been reported that Wnt/β-catenin signaling may play an important role in ovarian cancer development^19^, our initial analysis demonstrated low levels of endogenous Wnt/β-catenin activity in HeyA8 and SKOV3 cells. Subsequently, we sought to determine which, if any, cancer-associated pathways were modulated by monensin. A panel of the 11 cancer-associated pathways was used, as previously described^20^,^21^,^22^,^23^. When the Gaussia luciferase reporters for the 11 pathways and a constitutively active reporter pG2Luc were transfected into SKOV3 cells and treated with 0, 2 μM or 4 μM monensin for 48 h, it was found that Gaussia luciferase activities for the Elk1/SRF, AP1, NFκB and STAT reporters were significantly inhibited. A slight but apparent inhibition of Myc/Max reporter activity was also noted (Fig. 3C). We further tested if the inhibitory effect of monensin on Elk1/SRF, AP1, NFκB and STAT reporters was time- and/or dose-dependent. As shown in Fig. 3D, the Gaussia luciferase activities of these four reporters were effectively inhibited in SKOV3 cells in a dose and time-dependent fashion, and a significant inhibition on the four reporters was achieved at 72 h post monensin treatment (Fig. 3D). Similar reporter assay results were obtained with HeyA8 cells (data not shown). Given that the reporter assays suggested that monensin may target the growth factor signaling pathways, we evaluated expression levels of the proliferation related genes following treatment with monensin. Using our recently optimized touchdown-quantitative real-time PCR or TqPCR^24^, we found that expression of the five examined genes, EGFR, STAT3, c-Myc, Bcl-2 and cyclin D1, was effectively inhibited by monensin at 1 μM and 2 μM, respectively (Fig. 3E). Taken together, these results strongly suggest that monensin may exert its strong anti-proliferative activity by inhibiting growth factor receptor-induced signaling pathways involving the activation of receptor tyrosine kinases, JAK/STAT, MAPK, and/or NFκB downstream signaling mediators in ovarian cancer cells.

### Monensin effectively inhibits the expression of EGFR, but not IGF-1R, and synergizes with EGFR inhibitors in suppressing proliferation of human ovarian cancer cells

**Figure 4:**
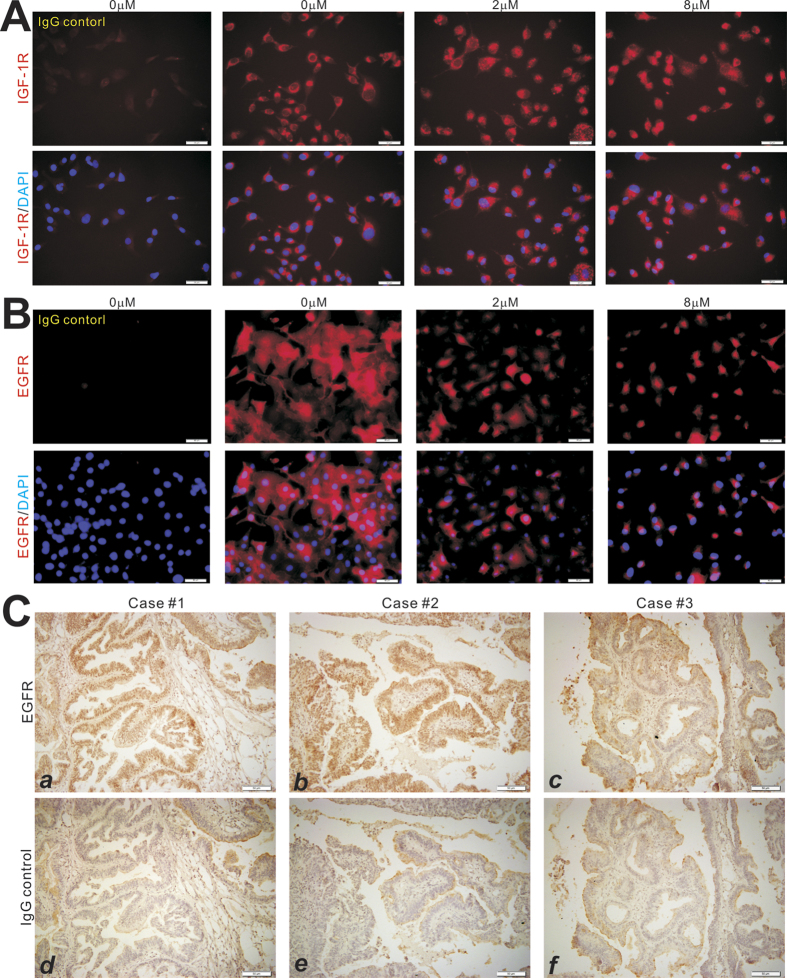
Monensin inhibits EGFR, but not IGF-1R, expression in human ovarian cancer cells. (**A**) Monensin does not affect IGF-1R expression in ovarian cancer cells. Subconfluent SKOV3 cells were treated with monensin at the indicated concentrations for 36 h, fixed and subjected to immunofluorescence staining with an anti-IGF-1R antibody. (**B**) Monensin suppresses EGFR expression in ovarian cancer cells. SKOV3 cells were treated with monensin at the indicated concentrations for 36 h, fixed and subjected to immunofluorescence staining with an anti-EGFR antibody. (**C**) EGFR is highly expressed in ovarian cancer tissues. Sections from three clinically diagnosed ovarian cancer samples were subjected to immunohistochemical staining using an anti-EGFR antibody. Control IgG was used as negative controls. Representative images are shown.

As EGFR and IGF-1R are two of the mostly commonly activated growth factor signaling pathways in human cancers^25^,^26^,^27^, we examined if monensin exerts its anti-proliferative activity by targeting either of these pathways. Subconfluent SKOV3 cells were treated with varying concentrations of monensin for 36 h. Expression of IGF-1R was examined by immunofluorescence staining. No significant changes in IGF-1R expression were observed, even with 8 μM monensin (Fig. 4A). However, under the same treatment condition, we found that the expression of EGFR was significantly inhibited by monensin at concentrations as low as 2 μM (Fig. 4B). These results are in concordance with previously mentioned qPCR results, which demonstrated decreased expression of EGFR in ovarian cancer cells (Fig. 3E).

To determine the clinical relevance of our findings, we further examined EGFR expression in ten patient samples of ovarian cancer. Seven of the ten cases had strong EGFR staining in cancerous cells relative to surrounding stromal cells and IgG controls, similar to the results shown in Fig. 4C (**panels** ***a,b vs. d,e***). The remaining three samples had weaker but detectable expression of EGFR in cancerous regions (Fig. 4C**, panels** ***c vs. f***). Taken together, these results strongly suggest that EGFR may serve as one of the important cellular targets of monensin, and may explain in part the anti-proliferative activity monensin demonstrates against ovarian cancer cells.

### Monensin synergizes with EGFR inhibitors in suppressing cell proliferation of human ovarian cancer cells

**Figure 5:**
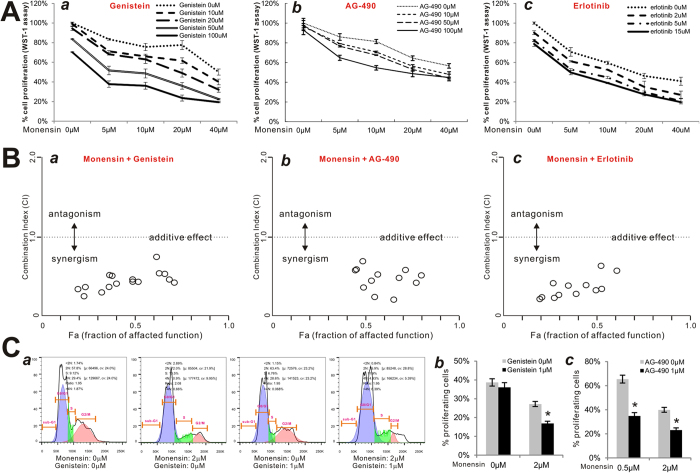
Monensin synergizes with EGFR inhibitors in suppressing cell proliferation of human ovarian cancer cells. (**A**) Monensin synergizes with EGFR inhibitors in suppressing cell proliferation in ovarian cancer cells. Subconfluent SKOV3 cells were treated with monensin and genestein (*a*), AG-490 (*b*), and erlotinib (*c*) at the indicated concentrations. At 24 h post treatment, WST-1 reagent was added to the culture medium and incubated for 1 h. WST-1 activities were measured at 440 nm. Assays were done in triplicate. (**B**) Synergy quantification between monensin and EGFR inhibitors. WST-1 assay data obtained from (**A**) were calculated for the combination index (CI) using the Chou-Talalay method^28^. Monensin was shown to have synergism (i.e., CI < 1) with genestein (*a*), AG-490 (*b*), and erlotinib (*c*). **(C)** Monensin acts synergistically with EGFR inhibitors in suppressing cell cycle progression. Subconfluent SKOV3 cells were treated with monensin and genestein or AG-490 the indicated concentrations for 24 h. Cells were collected, fixed, stained with Hoechst 33258, and subjected to FACS analysis (*a*). Percentages of cells in the S/M/G2 phases were tabulated and graphed (*b* & *c*). Each assay condition was done in triplicate. *p < 0.05 (combination group vs. single treatment group).

We analyzed if monensin exhibits any synergistic anti-proliferative effect with other tyrosine kinase inhibitors and clinically-used EGFR inhibitors. When exponentially growing SKOV3 cells were treated with various concentrations of both monensin and tyrosine kinase inhibitor genestein, cell proliferation rates significantly decreased with increasing concentrations of either monensin (0 to 40 μM) or genestein (0 to 100 μM) (Fig. 5A**, panel a**). Although similar results were obtained when tyrosine kinase inhibitor AG-490 (0 to 100 μM) was used with monensin, the magnitude of reduction in cell proliferation rate was slightly less than that obtained with equivalent concentrations of genestein (Fig. 5A**, panel** ***b***). Furthermore, low concentrations of the clinically-used EGFR inhibitor erlotinib (0 to 15 μM) caused significant decreases in cell proliferation in the presence of varied concentrations of monensin (Fig. 5A**, panel** ***c***). Quantitative calculations of the combination index (CI) using the Chou-Talalay method^28^ reveal that the CI values for monensin/genestein (Fig. 5B**, panel** ***a***), monensin/AG-490 (Fig. 5B**, panel** ***b***), and monensin/erlotinib (Fig. 5B**, panel** ***c***) are all less than 1.0, indicating that monensin exhibits strong synergistic effects with these agents to inhibit proliferation of ovarian cancer cells.

We further analyzed possible synergistic effects between monensin and these inhibitors on cell cycle progression and found that while monensin and genestein alone induce G1 arrest in ovarian cancer cells, the combination of these two agents significantly increased the percentage of cells arrested in G1 phase (Fig. 5C**, panels** ***a & b***). Similarly, AG-490 was shown to potentiate monensin-induced inhibition of cell cycle progression, although the maximal inhibition rate was less than that of equivalent concentrations of genestein (Fig. 5C **panel c**). These results demonstrate that monensin works synergistically with EGFR inhibitors to suppress ovarian cancer cell proliferation.

### Monensin synergizes with the chemotherapeutic drug oxaliplatin to inhibit cell proliferation and induce apoptosis of human ovarian cancer cells

**Figure 6:**
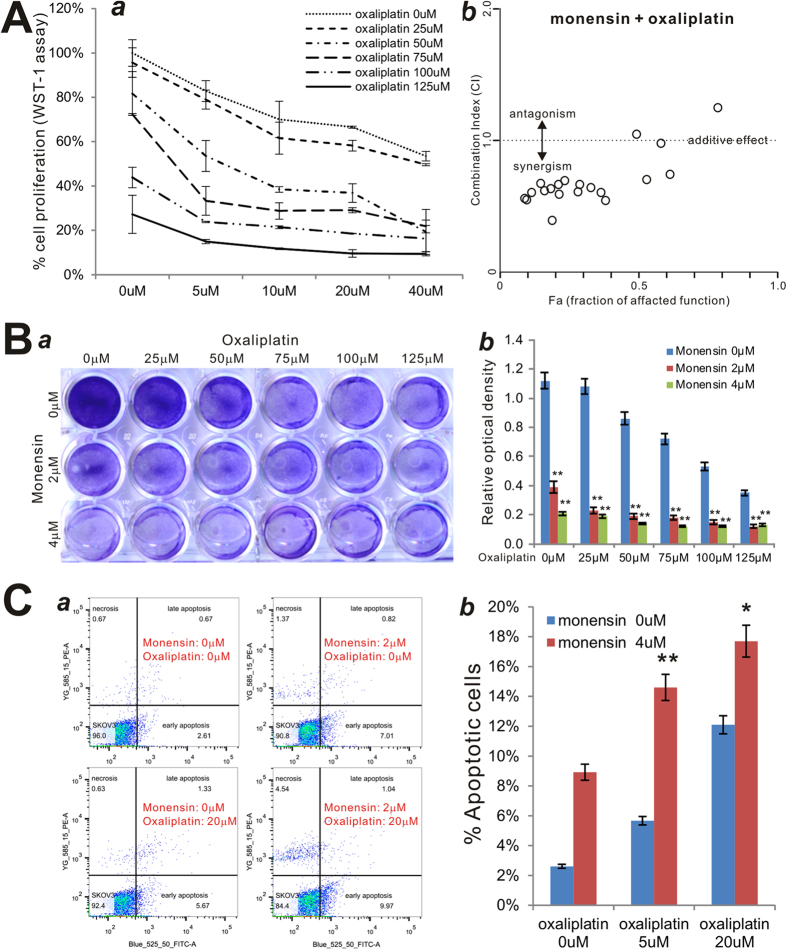
Monensin synergizes with oxaliplatin in inhibiting cell proliferation and inducing apoptosis of human ovarian cancer cells. (**A**) Synergism between monensin and oxaliplatin. SKOV3 cells were with monensin and oxaliplatin at the indicated concentrations. At 24 h post treatment, WST-1 reagent was added to the culture medium and incubated for 1 h. WST-1 activities were measured at 440 nm (*a*). Assays were done in triplicate. WST-1 assay data were calculated for the combination index (CI) using the Chou-Talalay method^28^. Monensin was shown to have synergism (i.e., CI < 1) with oxaliplatin (*b*). (**B**) Crystal violet staining assay. HeyA8 cells were treated with monensin and oxaliplatin at the indicated concentrations. At 72 h post treatment, cells were fixed and stained with crystal violet. Assay was done in triplicate, and representative images are shown (a). The crystal violet stained cells were dissolved in acetic acid and measured quantitatively for optical absorbance (b). **p < 0.01 (combination group vs. Oxaliplatin only treatment group). (**C**) Annexin-V apoptosis assay. SKOV3 cells were treated with monensin and oxaliplatin at the indicated concentrations. At 24 h post treatment, cells were collected and stained with Annexin V-FITC and propodium iodide, and subjected to flow cytometry (*a*). Average percentages of apoptotic cells were calculated and graphed (*b*). *p < 0.05, **p< 0.01 (combination group vs. single treatment group).

If monensin can be repurposed as an anti-ovarian cancer agent, it would be of significant if monensin can synergize with currently-used chemotherapeutic drugs such as oxaliplatin to inhibit cancer cell proliferation. When exponentially-proliferating SKOV3 cells were treated with varying concentrations of monensin or oxaliplatin, significant inhibition of cell proliferation was observed in a dose-dependent fashion (Fig. 6A**, panel a**). However, oxalipatin was shown to also potentiate monensin-induced inhibition of cell proliferation (Fig. 6A**, panel a**). Based on WST-1 assays, the calculated combination index using the Chou-Talalay method^28^ indicates that monensin demonstrates synergism with oxaliplatin (i.e., CI < 1) (Fig. 6A**, panel b**). This synergism was further confirmed via crystal violet staining assay qualitatively and quantitatively (Fig. 6B**, panels a,b**). Additionally, Annexin-V based apoptosis assay demonstrated that a combination of 2 μM monensin and 20 μM oxaliplatin reduces the viable cell population to 84.4% from 96% (no drug control), 90.8% (2 μM monensin only), and 92.4% (20 μM oxaliplatin only) (Fig. 6C**, panel a**). At a higher concentration of monensin (4 μM) oxaliplatin was shown to increase apoptosis even more effectively (Fig. 6C**, panel b**). Thus, these results indicate that monensin can synergize with oxaliplatin by inhibiting proliferation and inducing apoptosis of human ovarian cancer cells.

### Monensin inhibits xenograft tumor growth through inhibiting cell proliferation by possibly targeting EGFR signaling

**Figure 7:**
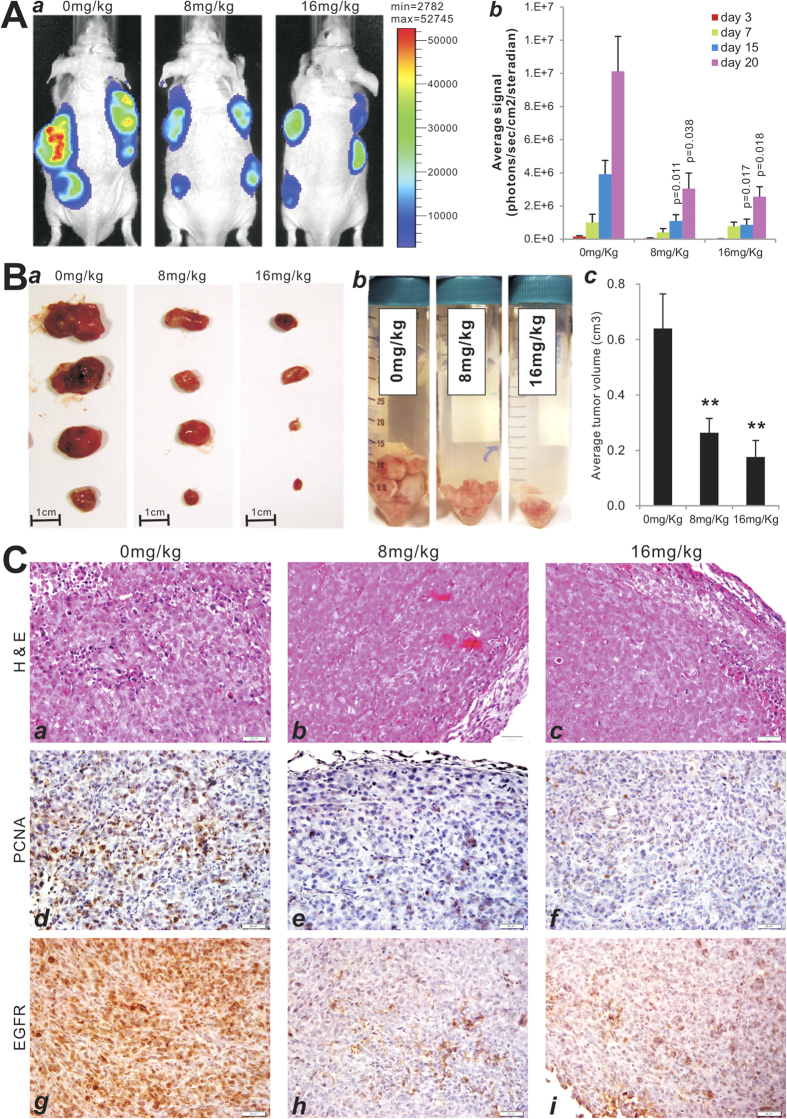
Monsensin effectively inhibits tumor growth in the xenograft model of human ovarian cancer cells. (**A**) Xenogen bioluminescence imaging of xenograft tumor growth. Firefly luciferase-labeled HeyA8 cells were injected into athymic nude mice subcutaneously. At three days post injection, the animals were treated with monensin (8 mg/kg, 16 mg/kg) or vehicle control. The mice were imaged at 3, 7, 15, and 20 days after treatment, and sacrificed at day 20. Representative images at day 20 are shown (*a*). The average signal for each group at different time points were calculated using the Xenogen’s Living Image analysis software (*b*). p-values are indicated in the graph. (**B**) Representative gross images of the retrieved tumor samples (*a*), accumulative tumor masses from each group (*b*), and the average tumor volume for each group (*c*). **p< 0.001 (monensin group vs. control group). **(C)** The retrieved tumor samples from each group were paraffin-embedded, sectioned and subjected to H&E staining (*a-c*). Sections were further subjected to immunohistochemical staining using anti-PCNA (*d-f*) or anti-EGFR (*g*-*i*) antibody. Control IgGs were used as negative controls (not shown). Representative images are shown.

We tested the *in vivo* anti-cancer activity of monensin in the xenograft tumor model of human ovarian cancers. Exponentially growing firefly luciferase-tagged HeyA8 ovarian cancer cells were injected subcutaneously into the flanks of athymic nude mice. At three days post-injection, the animals were treated with two doses of monensin (8 mg/kg body weight and 16 mg/kg body weight) or vehicle control. Tumor growth was monitored by using Xenogen bioluminescence imaging for up to 20 days post-treatment (Fig. 7A **panel a**). Quantitative analysis of Xenogen imaging data indicated that monensin effectively inhibited tumor growth at 15 and 20 days after treatment at both doses when compared with the vehicle control group (Fig. 7A **panel b**). At the study endpoint, the tumor masses were retrieved, and the control group had significantly larger individual tumors (Fig. 7B **panel a**), a larger bulk tumor volume (Fig. 7B **panel b**), and higher average tumor volume (Fig. 7B **panel c**) when compared to treatment groups. These results were consistent with the results obtained from Xenogen imaging analysis.

Histologic evaluation was also carried out on the retrieved tumor samples. H&E staining revealed that monensin-treated tumor samples exhibited extensive necrosis relative to samples of the control group (Fig. 7C **panels** ***a vs. b,c***). Immunohistochemical staining with the proliferating cell nuclear antigen (PCNA) antibody demonstrated a significant decrease in the number of PCNA positive cells in monensin treatment groups, especially with higher monensin dose (16 mg/kg bw), relative to the control group (Fig. 7C **panels** ***d vs. e,f***). We further examined the EGFR expression status in the retrieved tumor samples and found that monensin treatment groups exhibited drastically diminished EGFR expression relative to the control group (Fig. 7C **panels** ***g vs. h,i***), consistent with earlier findings (Fig. 4B,C) and strengthening the proposition that EGFR may serve as a key cellular target explaining anti-proliferative effects of monensin in human ovarian cancer cells.

## Discussion

### Monensin may be repurposed as an effective anticancer agent for human ovarian cancer

Although the 5-year survival rate for ovarian cancer patients has improved over the past two decades with improvements in surgical technique and empiric advances in cytotoxic chemotherapy regimens, the overall cure rate remains approximately 30%^1^,^6^. Ultimately, most patients experience recurrence within 12–24 months and expire secondary to progressively treatment-resistant disease. Thus, there is a critical need to develop more effective and novel therapies to treat ovarian cancers. Our results have demonstrated that monensin acts synergistically with tyrosine kinase and EGFR inhibitors, as well as currently-utilized agents such as oxaliplatin, to suppress proliferation of ovarian cancer cells. Our results are encouraging and suggest a utility for repurposing monensin as a part of combination chemotherapy strategy for the clinical management of ovarian cancer.

Monensin is a polyether ionophore antibiotic secreted by the bacteria *Streptomyces cinnamonensis*^9^,^10^. Monensin can freely pass across the lipid bilayer of the cytoplasmic membrane or cellular organelles transporting ions along by passive diffusion^9^,^10^. Monensin has been shown to have a positive safety profile in veterinary medicine; it has been used in cattle and poultry feed for nearly 50 years^9^,^10^. Several earlier studies indicate that monensin exhibited cytotoxic effects on several types of cancer cells, including renal cancer, colon cancer, myeloma, lymphoma, and prostate cancer cell lines^10^,^11^,^12^,^13^,^14^,^15^,^16^,^17^,^18^. A recent study showed that malignant cell lines are more than 20-fold more sensitive to monensin than their nonmalignant counterparts^12^, indicating that monensin may target cancer cells more preferentially than most conventionally-used cytotoxic chemotherapy drugs.

### Monensin may exert anticancer activity by targeting multiple signaling pathways

Given that monensin has a favorable safety profile and acts effectively at low micromolar concentrations, further investigation of the detailed mechanism underlying its mode of action is warranted. As a carboxylic Na^+^/H^+^ ionophore, monensin has been noted to exert significant effects on function and activity of the Golgi apparatus and the intracellular trafficking and processing of endocytosis^29^. Earlier studies in fact utilized monensin conjugates or liposomes to deliver therapeutic monoclonal antibodies, immunotoxins, or chemotherapy drugs^30^,^31^,^32^,^33^,^34^,^35^. Mechanistically, earlier studies indicate that monensin was shown to decrease levels of CDK6, cyclin D1 and cyclin A and to induce apoptosis-associated changes in Bax, caspase-3, caspase-8 and mitochondria transmembrane potential in several human cancer cell lines^13^,^14^,^15^,^16^,^17^. It has been reported that mitochondrial damage is an early event of monensin-induced cell injury in cultured fibroblasts L929^36^. Monensin was shown to be a potent inducer of oxidative stress and inhibitor of androgen signaling leading to apoptosis in prostate cancer cells^12^. More recently, monensin was shown to inhibit canonical Wnt signaling in human colorectal cancer cells and to suppress tumor growth in multiple intestinal neoplasia mice^18^. Collectively, these reports strongly suggest that monensin may target cancer cells through a diverse set of mechanisms.

We analyzed the effect of monensin on 11 cancer-associated pathways and found that monensin inhibits the reporter activities for the Elk1/SRF, AP1, NFκB and STAT pathways, and to a lesser extent the Myc/Max reporter activity. Furthermore, the expression of the five examined genes, EGFR, STAT3, c-Myc, Bcl-2 and cyclin D1, was effectively inhibited by monensin. Thus, our results strongly suggest that monensin may exert its strong anti-proliferative activity by inhibiting growth factor receptor-induced signaling pathways, which involve the activation of receptor tyrosine kinases, JAK/STAT, MAPK, and/or NFκB downstream signaling mediators in ovarian cancer cells. Interestingly, EGFR mutations have rarely been reported thus far in ovarian cancer, but the receptor expression is readily detectable. EGFR inhibitors gefitinib and erlotinib were shown to stabilize disease in up to 44% of patients with ovarian cancer^37^,^38^, although the inhibitory activity of these inhibitors may be mitigated by the remarkably activated downstream mediators such as PI3K and MAPK signaling^1^. Nonetheless, two recent studies indicate that monensin affects the endocytic recycling pathway for EGFR^10^,^39^. Our immunostaining results also revealed that EGFR level was significantly suppressed upon monensin treatment. The reported findings and our results may at least partially explain the synergistic effects between monensin and EGFR inhibitors in suppressing cell proliferation of ovarian cancer cells. Nonetheless, further investigations into the molecular mechanisms through which monensin exerts its anti-cancer activity are warranted.

Monensin (or Rumensin) is FDA approved for veterinary use (beef cattle, chickens, dairy cattle, turkeys, veal), and is the most potent feed ingredient available that kills coccidia parasites. For the prevention and control of coccidiosis, the animals are fed at a rate to provide 0.14 to 0.42 mg/lb of body weight/d of monensin up to a maximum of 200 mg/herd/day. The *in vivo* use of monensin for its anticancer activity has been reported and dose ranges were similar to that we used in this study. Nonetheless, a full scale of pre-clinical pharmacokinetics and toxicology for monensin remains to be carried out so that monensin can be moved forward as a clinically repurposed anticancer agent.

In summary, we investigated the potential of repurposing monensin as an anti-cancer agent for human ovarian cancer. Our results revealed that monensin effectively inhibits cell proliferation, cell migration, and cell cycle progression, and induces apoptosis of human ovarian cancer cells. Monensin was shown to target multiple cancer-related signaling pathways such as Elk1/SRF, AP1, NFκB and STAT, and suppresses EGFR expression in ovarian cancer cells. Monensin was further shown to act synergistically with EGFR inhibitors and the chemotherapeutic drug oxaliplatin to inhibit cell proliferation and induce apoptosis of human ovarian cancer cells. The *in vivo* xenograft studies further confirm that monensin effectively inhibits xenograft tumor growth by inhibiting cell proliferation through targeting EGFR signaling. Thus, our results strongly suggest that monensin may be repurposed as an anti-ovarian cancer agent. Future studies should be directed towards testing monensin’s anti-cancer efficacy in preclinical and clinical studies.

## Materials and Methods

### Cell culture and chemicals

Human ovarian cancer cell lines SKOV3 and HeyA8 were generously provided by Dr. Ernest Lengyel. HEK-293 cells were purchased from ATCC (Manassas, VA). The cells were maintained in complete Dulbecco’s Modified Eagle’s Medium (DMEM) containing 10% fetal bovine serum (FBS, Invitrogen, Carlsbad, CA), 100 units of penicillin and 100 μg of streptomycin at 37 °C in 5% CO_2_ as described^40^,^41^,^42^,^43^,^44^,^45^. Chemicals monensin (aka, rumensin), genestein (aka, CI 75610, genistein, genisteol, or genisterin), AG-490 (aka, Tyrphostin AG-490), or erlotinib (aka, Tarceva), were purchased from Cayman Chemical (Ann Arbor, MI). Unless indicated otherwise, all chemicals were purchased from Sigma-Aldrich (St. Louis, MO) or Fisher Scientific (Pittsburgh, PA).

### Crystal violet cell viability assay

Crystal violet staining assay was conducted as described^40^,^41^,^42^,^43^,^44^,^45^,^46^,^47^,^48^,^49^,^50^,^51^. Briefly, subconfluent HeyA8 and SKOV3 cells were treated with varied concentrations of monensin or ethanol control. At 72 h after treatment, cells were washed with PBS and stained with 0.5% crystal violet/formalin solution at room temperature for 20–30 min. The stained cells were washed with tape water and air dried for taking macrographic images^42^. For quantitative measurement, the stained cells were dissolved in 10% acetic acid at room temperature for 20 min with shaking, followed by measuring absorbance at 570–590 nm^22^,^41^,^42^.

### Viable cell counting assay

Viable cells were counted with Trypan blue exclusion staining assay as described^52^. Briefly, subconfluent SKOV3 and HeyA8 cells were treated with monensin at the indicated concentrations or vehicle control. At 48 h and 72 h, cella were collected by trypsin dissociation, and stained with Trypan blue (final concentration at 0.1% Trypan blue). Unstained viable cells were counted under a bright field microscope. Each assay condition was done in triplicate.

### WST-1 cell proliferation assay

Cell proliferation was assessed by using Premixed WST-1 Reagent (Clontech, Mountain View, CA) as described^42^. Briefly, subconfluent SKOV3 and HeyA8 cells seeded in 96-well plates were treated with monensin and/or other drugs at the varied concentrations for 24 h or 48 h. The Premixed WST-1 Reagent was added to each well, followed by an incubation at 37 °C for 30 to 60 min and reading at 440 nm using the microplate reader (BioTek EL800, Winooski, VT). Each assay condition was done in triplicate.

### Cell wounding/migration assay

Cell wounding/migration assay was performed as described^45^,^53^. Briefly, exponentially growing ovarian cancer cells were seeded in 6-well cell culture plates and allowed to reach approximately 90% confluence. Then, the monolayer cells were wounded with sterile micro-pipette tips. At various time points, the wound healing status at the approximately same locations was recorded under bright field microscopy. Each assay condition was done in triplicate.

### Apoptosis analysis (Hoechst 33258 staining)

As previously described^22^,^23^,^45^,^53^, exponentially growing HeyA8 and SKOV3 cells were treated with varied concentrations of monensin or ethanol control. At 24 h post treatment, cells were collected, fixed and stained with the Magic Solution (10× stock: 0.5% NP-40, 3.4% formaldehyde, 10 μg/ml Hoechst 33258, in PBS). Apoptotic cells were examined and recorded under a fluorescence microscope. Each assay condition was done in triplicate. The results were repeated at least in three independent batches of experiments. The average numbers of apoptotic cells were calculated by counting apparent apoptotic cells in at least ten random fields at 100× magnification for each assay condition.

### Apoptosis analysis (Annexin V-FITC flow cytometry

The annexin V staining apoptosis assay was performed as previously described^22^,^45^,^53^. Briefly, exponentially growing SKOV3 cells were seeded in 6-well plates and treated with monensin and/or other drugs at the indicated concentrations. At 24 h post treatment, cells were trypsinized, washed with PBS, resuspended in Annexin V Binding Buffer at a density of 10^6^ cells/ml, and stained with Annexin V-FITC (BD Pharmingen, San Jose, CA) and propidium iodide for 15 min at room temperature under a light-proof condition. The stained cells were subjected to flow cytometry analysis using the BD FACSCalibur-HTS. The acquired flow cytometry data were analyzed by using the FlowJo v10.0 software. Each assay condition was done in triplicate.

### Cell cycle analysis

The exponentially growing HeyA8 and SKOV3 cells were seeded in 6-well plates at sub-confluence and treated with varied concentrations of monensin or ethanol control. At 24 h or 48 h post treatment, cells were collected, fixed and stained with the Magic Solution for 30 min. The stained cells were subjected to flow cytometry analysis using the BD FACSCalibur-HTS. The acquired flow cytometry data were analyzed with the FlowJo v10.0 software. Each assay condition was done in triplicate.

### Construction and amplification of recombinant adenovirus expressing Wnt3A or GFP

Recombinant adenovirus expressing Wnt3A was constructed by using the AdEasy system as described^54^,^55^,^56^,^57^. Briefly, the mouse Wnt3A coding region was PCR amplified and subcloned into an adenoviral shuttle vector, and used to generate and amplify recombinant adenovirus in HEK-293 or 293pTP cells^58^. The resulting adenovirus was designated as AdWnt3A, which also expresses GFP^59^,^60^,^61^,^62^. An analogous adenovirus expressing only GFP (AdGFP) was used as a control^63^,^64^. For all adenoviral infections, polybrene (4-8μg/ml) was added to enhance infection efficiency as previously reported^44^.

### Immunofluorescence staining

The immunofluorescence staining assays were carried out as previously described^23^,^45^. Briefly, for β-catenin staining the cells were first infected with AdWnt3A or AdGFP for 16 h, replated into 24-well plates, and then treated with monensin at varied concentrations or vehicle control. For IGF-1R and EGFR staining assays, subconfluent SKOV3 cells were treated with monensin at varied concentrations or vehicle control. At 36 h post treatment, the cells were fixed and subjected to immunofluorescence staining with antibody against β-catenin (Santa Cruz Biotechnology, Santa Cruz, CA), IGF-1R (Santa Cruz Biotechnology), or EGFR (Santa Cruz Biotechnology). Control IgG and minus primary antibodies were used as negative controls.

### Immunohistochemical (IHC) staining

The use of human ovarian cancer tissue samples was approved by the Institutional Ethic Committee of Chongqing Medical University. The archived ovarian cancer samples were delinked from the patients’ private information and approved for IHC use with the waived informed consent according to the United States National Institutes of Health’s guidelines involving human subjects. Ten cases of human ovarian cancer samples were obtained from the Department of Obstetrics and Gynecology, the First Affiliated Hospital of Chongqing Medical University, Chongqing, China. The IHC staining was performed as described^45^,^46^,^52^,^65^. Briefly, sections of the paraffin-embedded tissue blocks were deparaffinized, rehydrated, and subjected to immunohistochemical staining with anti-EGFR or anti-PCNA (Santa Cruz Biotechnology) antibody. Control IgG and minus primary antibodies were used as negative controls.

### Cell transfection and luciferase reporter assay

For the TOP-Luc firefly luciferase (FLuc) reporter assay^21^,^53^,^66^,^67^, the subconfluent SKOV3 cells were first transfected with TOP-Luc reporter plasmid using Lipofectamine (Invitrogen, Carlsbad, CA). At the end of transfection, the cells were infected with Ad-Wnt3A or AdGFP for 16 h, followed by addition of monensin with varied concentrations for another 48 h. Cells were lysed and subjected to luciferase activity assays using Promega’s firefly Luciferase Assay System. Each assay condition was done in triplicate.

The Gaussia luciferase (GLuc) reporter assay was carried out as described^20^,^44^,^67^,^68^. The tested 11 cancer-relate signaling pathway reporters were homemade and previously described 20, including NFAT, HIF-1, E2F/DP1, Elk1/SRF, AP1, NFκB, Smad, STAT1/2, RBP-Jκ, CREB, Myc/Max reporters. A constitutively active reporter pG2Luc was used as a control. Experimentally, subconfluent SKOV3 cells were seeded in 25 cm^2^ culture flasks and transfected with 3.0 μg per flask of the 12 reporter plasmids using Lipofectamine (Invitrogen). At 16 h post transfection, cells were replated in 12-well plates and treated with various concentrations of monensin or ethanol control. At 24 h, 48 h or 72 h post treatment, culture media were taken and subjected to Gaussia luciferase assays using the BioLux Gaussia Luciferase Assay Kit (New England Biolabs). Each assay condition was done in triplicate. Luciferase activity was normalized by total cellular protein concentrations among the samples.

### Total RNA isolation and touchdown-quantitative real-time PCR (TqPCR) analysis

Subconfluent ovarian cancer cells were treated with varied concentrations of monesin for 48 h. Total RNA was isolated from the treated cells by using TRIZOL Reagents (Invitrogen) and subjected to reverse transcription reactions with hexamer and M-MuLV reverse transcriptase (New England Biolabs, Ipswich, MA). Such cDNA products were used as PCR templates. The qPCR primers were designed by using Primer3 program^69^ and used to amplify the genes of interest (Supplemental Table 1). The TqPCR were carried out by using the SYBR Green-based qPCR analysis on a CFX-Connect unit (Bio-Rad Laboratories, Hercules, CA), as described^24^. The qPCR reactions were done in triplicate. GAPDH was used as a reference gene.

### Chou–Talalay drug combination index analysis

The combination effects between monensin and the EGFR inhibitors (Genestein, AG-490, or Erlotinib), or oxaliplatin were calculated with the Chou–Talalay method^28^. The dose effect curves of each drug alone, and in combination, were generated by WST-1 assay. These data were analyzed with the CompuSyn software (ComboSyn, Inc.). The calculated combination index (CI) theorem of Chou-Talalay offers quantitative definition for additive effect (CI = 1), synergism (CI < 1), and antagonism (CI > 1) in drug combinations^28^.

### Xenogratft tumors of human ovarian cancer cells

The use and care of animals were approved by the Institutional Animal Care and Use Committee at The University of Chicago. All experimental procedures were carried out in accordance with the approved guidelines. Briefly, HeyA8 stably labeled with firefly luciferase (HeyA8-FLuc) was constructed with *piggyBac* system^41^,^43^,^70^. Exponentially growing HeyA8-FLuc cells were collected, resuspended at 10^7^ cells/ml and injected subcutaneously into the flanks of athymic nude mice (Harlan Laboratories, 6–8 week old, male, 10^6^ cells per injection, and 4 sites per mouse). The mice were divided into three groups (n = 5 per group). At three days post injection, the animals were treated with various doses of monensin (8 mg or 16 mg/kg body weight) or vehicle control (ethanol) intraperitoneally once every two days. Tumor growth was monitored by whole body bioluminescence imaging using Xenogen IVIS 200 Imaging System at days 3, 7, 15, and 20 after treatment. The mice were sacrificed at 3 weeks and subcutaneous tumor masses were retrieved for histologic evaluation and immunohistochemistry.

### Statistical analysis

The quantitative assays were performed in triplicate and/or repeated three times. Data were expressed as mean ± D. Statistical significances were determined by one-way analysis of variance and the student’s *t* test. A value of *p* < 0.05 was considered statistically significant.

## Additional Information

**How to cite this article**: Deng, Y. *et al.* Antibiotic monensin synergizes with EGFR inhibitors and oxaliplatin to suppress the proliferation of human ovarian cancer cells. *Sci. Rep.* **5**, 17523; doi: 10.1038/srep17523 (2015).
